# 2-Methyl-6-[2-(trifluoro­meth­yl)phenyl­imino­meth­yl]phenol

**DOI:** 10.1107/S1600536809044560

**Published:** 2009-10-31

**Authors:** Hasan Tanak, Metin Yavuz, Orhan Büyükgüngör

**Affiliations:** a Department of Physics, Faculty of Arts & Science, Ondokuz Mayıs University, TR-55139 Kurupelit-Samsun, Turkey

## Abstract

The title compound, C_15_H_12_F_3_NO, is a Schiff base which adopts the phenol–imine tautomeric form in the solid state. The dihedral angle between the aromatic rings is 38.79 (5)°. The mol­ecular structure is stabilized by an intra­molecular O—H⋯N hydrogen bond, which generates an *S*(6) ring. In addition, there is an intra­molecular short C—H⋯F contact.

## Related literature

For the biological properties of Schiff bases, see: Barton *et al.* (1979 ▶); Layer (1963 ▶); Ingold (1969 ▶) Taggi *et al.* (2002 ▶); Aydoğan *et al.* (2001 ▶). Schiff base compounds can be classified by their photochromic and thermochromic characteristics, see: Cohen *et al.* (1964 ▶); Moustakali-Mavridis *et al.* (1978 ▶). For the graph-set description of hydrogen bonds, see: Bernstein *et al.* (1995 ▶. For a related structure, see: Temel *et al.* (2007 ▶).
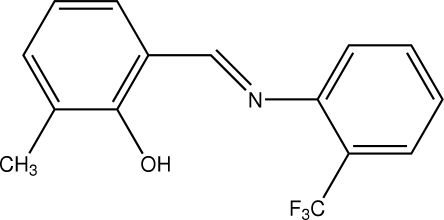


## Experimental

### 

#### Crystal data

C_15_H_12_F_3_NO
*M* *_r_* = 279.26
Orthorhombic, 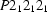

*a* = 8.1634 (3) Å
*b* = 11.8810 (6) Å
*c* = 13.4469 (7) Å
*V* = 1304.21 (11) Å^3^
*Z* = 4
Mo *K*α radiation
μ = 0.12 mm^−1^
*T* = 293 K
0.73 × 0.51 × 0.37 mm

#### Data collection

Stoe IPDS II diffractometer
Absorption correction: integration (*X-RED32*; Stoe & Cie, 2002 ▶) *T*_min_ = 0.943, *T*_max_ = 0.970
14752 measured reflections
1565 independent reflections
1396 reflections with *I* > 2σ(*I*)
*R*_int_ = 0.030

#### Refinement

*R*[*F* ^2^ > 2σ(*F* ^2^)] = 0.031
*wR*(*F* ^2^) = 0.081
*S* = 1.07
1565 reflections
187 parameters
H atoms treated by a mixture of independent and constrained refinement
Δρ_max_ = 0.09 e Å^−3^
Δρ_min_ = −0.15 e Å^−3^

### 

Data collection: *X-AREA* (Stoe & Cie, 2002 ▶); cell refinement: *X-AREA*; data reduction: *X-RED32* (Stoe & Cie, 2002 ▶); program(s) used to solve structure: *SHELXS97* (Sheldrick, 2008 ▶); program(s) used to refine structure: *SHELXL97* (Sheldrick, 2008 ▶); molecular graphics: *ORTEP-3 for Windows* (Farrugia, 1997 ▶); software used to prepare material for publication: *WinGX* (Farrugia, 1999 ▶).

## Supplementary Material

Crystal structure: contains datablocks I, global. DOI: 10.1107/S1600536809044560/bt5114sup1.cif

Structure factors: contains datablocks I. DOI: 10.1107/S1600536809044560/bt5114Isup2.hkl

Additional supplementary materials:  crystallographic information; 3D view; checkCIF report

## Figures and Tables

**Table 1:** Hydrogen-bond geometry (Å, °)

*D*—H⋯*A*	*D*—H	H⋯*A*	*D*⋯*A*	*D*—H⋯*A*
O1—H1⋯N1	0.93 (3)	1.77 (3)	2.619 (2)	151 (3)
C13—H13⋯F3	0.93	2.36	2.694 (3)	101

**Fig. 1.**
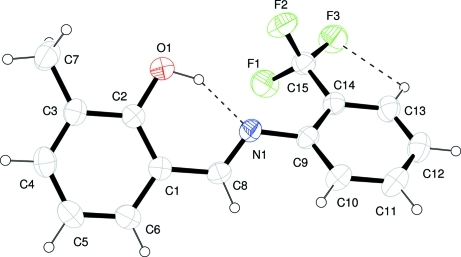
The molecular structure of the title compound, showing the atom-numbering scheme and 30% probability diplacement ellipsoids.

## supplementary crystallographic information

### Comment

Schiff bases, *i.e.*, compounds having a double C=N bond, are used as starting materials in the synthesis of important drugs, such as antibiotics and antiallergic, antiphlogistic, and antitumor substances (Barton *et al.*, 1979; Layer, 1963; Ingold 1969). On the industrial scale, they have a wide range of applications, such as dyes and pigments (Taggi *et al.*, 2002). Schiff bases have also been employed as ligands for the complexation of metal ions (Aydoğan *et al.*, 2001). There are two characteristic properties of Schiff bases, *viz*. Photochromism and thermochromism (Cohen *et al.*, 1964). In general, Schiff bases display two possible tautomeric forms, the phenol-imine (OH) and the keto-amine (NH) forms. Depending on the tautomers, two types of intramolecular hydrogen bonds are observed in Schiff bases: O—H···N in phenol-imine and N—H···O in keto-amine tautomers.

In the title compound (Fig. 1), the molecular structure is not planar. The dihedral angle between the aromatic ring systems [C1/C6 and C9/C14] is 38.79 (5)°. It is also known that Schiff bases may exhibit thermochromism depending on the planarity or non-planarity, respectively (Moustakali-Mavridis *et al.*, 1978).The O—H and C=N bond lengths confirm the phenol-imine form of the title compound. These distances agree with the corresponding distances in (*E*)-3-[2-(Trifluoromethyl)phenyliminomethyl]-benzene-1,2-diol (Temel *et al.*, 2007), which is related structure. The imine group is coplanar with the C1—C6 aromatic ring system as it can be shown by the C2—C1—C8—N1 torsion angle is 1.67 (19)°.

The molecular structure is stabilized by intramolecular hydrogen bonds. An intramolecular O1—H1···N1 hydrogen bond (Fig. 1) generates a six-membered ring, producing an S(6) ring motif (Bernstein *et al.*, 1995), resulting in approximate planarity of the molecular skeleton [O···N= 2.6187 (16) Å]. The crystal structure is further stabilized by intramolecular C—H···F hydrogen bond, namely C13—H13···F3. And also details of the hydrogen bond is shown in Table 1.

### Experimental

A solution of 3-methylsalicylaldehyde (0.0233 g, 0.1711 mmol) in ethanol (10 ml) was added to a solution of 2-Triflouromethylaniline (0.0275 g, 0.1711 mmol) in ethanol (20 ml). The reaction mixture was stirred for 2 h underreflux. Single crystals suitable for X-ray analysis were obtained from ethylalcohol by slow evaporation (yield 69%; m.p.408–410 K).

### Refinement

C-bound H atoms were positioned geometrically and refined using a riding model, with C—H = 0.93–0.96 Å and *U*_iso_(H) = 1.2–1.5*U*_eq_(C). The position of the H1 atom was obtained from a difference map and this atom was refined freely. Friedel pairs were merged in the final refinement because   the value of the absolute structure parameter (Flack, 1983) is meaningless.

### Crystal data

**Table:**

C_15_H_12_F_3_NO	*F*(000) = 576
*M**_r_* = 279.26	*D*_x_ = 1.422 Mg m^−^^3^
Orthorhombic, *P*2_1_2_1_2_1_	Mo *K*α radiation, λ = 0.71073 Å
Hall symbol: P 2ac 2ab	Cell parameters from 19471 reflections
*a* = 8.1634 (3) Å	θ = 1.5–28.0°
*b* = 11.8810 (6) Å	µ = 0.12 mm^−^^1^
*c* = 13.4469 (7) Å	*T* = 293 K
*V* = 1304.21 (11) Å^3^	Prism, light yellow
*Z* = 4	0.73 × 0.51 × 0.37 mm

### Data collection

**Table:**

Stoe IPDS II diffractometer	1565 independent reflections
Radiation source: fine-focus sealed tube	1396 reflections with *I* > 2σ(*I*)
graphite	*R*_int_ = 0.030
Detector resolution: 6.67 pixels mm^-1^	θ_max_ = 26.5°, θ_min_ = 2.3°
rotation method scans	*h* = −10→10
Absorption correction: integration (*X-RED32*; Stoe & Cie, 2002)	*k* = −14→14
*T*_min_ = 0.943, *T*_max_ = 0.970	*l* = −16→16
14752 measured reflections	

### Refinement

**Table:**

Refinement on *F*^2^	Primary atom site location: structure-invariant direct methods
Least-squares matrix: full	Secondary atom site location: difference Fourier map
*R*[*F*^2^ > 2σ(*F*^2^)] = 0.031	Hydrogen site location: inferred from neighbouring sites
*wR*(*F*^2^) = 0.081	H atoms treated by a mixture of independent and constrained refinement
*S* = 1.07	*w* = 1/[σ^2^(*F*_o_^2^) + (0.0562*P*)^2^ + 0.0179*P*] where *P* = (*F*_o_^2^ + 2*F*_c_^2^)/3
1565 reflections	(Δ/σ)_max_ < 0.001
187 parameters	Δρ_max_ = 0.09 e Å^−^^3^
0 restraints	Δρ_min_ = −0.15 e Å^−^^3^

### Special details

**Table:**

Experimental. 270 frames, detector distance = 100 mm
Geometry. All e.s.d.'s (except the e.s.d. in the dihedral angle between two l.s. planes) are estimated using the full covariance matrix. The cell e.s.d.'s are taken into account individually in the estimation of e.s.d.'s in distances, angles and torsion angles; correlations between e.s.d.'s in cell parameters are only used when they are defined by crystal symmetry. An approximate (isotropic) treatment of cell e.s.d.'s is used for estimating e.s.d.'s involving l.s. planes.
Refinement. Refinement of *F*^2^ against ALL reflections. The weighted *R*-factor *wR* and goodness of fit *S* are based on *F*^2^, conventional *R*-factors *R* are based on *F*, with *F* set to zero for negative *F*^2^. The threshold expression of *F*^2^ > σ(*F*^2^) is used only for calculating *R*-factors(gt) *etc*. and is not relevant to the choice of reflections for refinement. *R*-factors based on *F*^2^ are statistically about twice as large as those based on *F*, and *R*- factors based on ALL data will be even larger.

### Fractional atomic coordinates and isotropic or equivalent isotropic displacement parameters (Å^2^)

**Table:**

	*x*	*y*	*z*	*U*_iso_*/*U*_eq_	
C1	0.5950 (2)	0.12366 (13)	0.48589 (13)	0.0521 (4)	
C2	0.7604 (2)	0.09829 (14)	0.46506 (13)	0.0521 (4)	
C3	0.8634 (3)	0.05379 (15)	0.53818 (15)	0.0589 (5)	
C4	0.7966 (3)	0.03487 (16)	0.63136 (16)	0.0669 (5)	
H4	0.8626	0.0039	0.6806	0.080*	
C5	0.6363 (3)	0.06007 (18)	0.65389 (16)	0.0725 (6)	
H5	0.5960	0.0471	0.7176	0.087*	
C6	0.5366 (3)	0.10426 (16)	0.58227 (15)	0.0653 (5)	
H6	0.4285	0.1217	0.5977	0.078*	
C7	1.0381 (3)	0.0287 (2)	0.51468 (19)	0.0804 (6)	
H7A	1.0972	0.0979	0.5064	0.121*	
H7B	1.0440	−0.0144	0.4544	0.121*	
H7C	1.0857	−0.0137	0.5682	0.121*	
C8	0.4855 (2)	0.16517 (13)	0.41041 (14)	0.0536 (4)	
H8	0.3783	0.1826	0.4280	0.064*	
C9	0.4144 (2)	0.21161 (13)	0.24725 (14)	0.0525 (4)	
C10	0.2558 (3)	0.16864 (15)	0.24564 (17)	0.0638 (5)	
H10	0.2227	0.1181	0.2945	0.077*	
C11	0.1481 (3)	0.20022 (18)	0.1726 (2)	0.0757 (6)	
H11	0.0427	0.1705	0.1720	0.091*	
C12	0.1946 (3)	0.27551 (18)	0.10000 (19)	0.0747 (6)	
H12	0.1202	0.2976	0.0514	0.090*	
C13	0.3510 (3)	0.31792 (17)	0.09962 (16)	0.0661 (5)	
H13	0.3827	0.3681	0.0502	0.079*	
C14	0.4618 (2)	0.28640 (13)	0.17241 (14)	0.0541 (4)	
C15	0.6310 (3)	0.33225 (16)	0.17122 (15)	0.0625 (5)	
N1	0.53021 (19)	0.17904 (11)	0.31975 (11)	0.0530 (3)	
O1	0.82427 (18)	0.11539 (13)	0.37360 (11)	0.0671 (4)	
F1	0.67184 (18)	0.38548 (12)	0.25515 (11)	0.0888 (4)	
F2	0.74444 (17)	0.25364 (12)	0.15746 (12)	0.0872 (4)	
F3	0.65390 (18)	0.40802 (13)	0.09842 (12)	0.0930 (5)	
H1	0.738 (4)	0.141 (2)	0.335 (2)	0.097 (9)*	

### Atomic displacement parameters (Å^2^)

**Table:**

	*U*^11^	*U*^22^	*U*^33^	*U*^12^	*U*^13^	*U*^23^
C1	0.0552 (10)	0.0460 (7)	0.0550 (9)	−0.0031 (7)	0.0000 (8)	−0.0035 (7)
C2	0.0568 (10)	0.0468 (7)	0.0528 (9)	−0.0038 (7)	−0.0010 (8)	−0.0033 (7)
C3	0.0606 (11)	0.0518 (8)	0.0644 (11)	−0.0035 (8)	−0.0118 (9)	−0.0052 (8)
C4	0.0800 (15)	0.0577 (9)	0.0631 (11)	−0.0075 (9)	−0.0180 (11)	0.0033 (8)
C5	0.0891 (16)	0.0757 (11)	0.0528 (11)	−0.0107 (11)	0.0015 (11)	0.0036 (9)
C6	0.0685 (12)	0.0689 (10)	0.0584 (10)	−0.0042 (10)	0.0059 (10)	−0.0013 (9)
C7	0.0621 (13)	0.0903 (14)	0.0889 (16)	0.0081 (11)	−0.0139 (13)	−0.0037 (13)
C8	0.0492 (10)	0.0487 (7)	0.0630 (10)	0.0005 (7)	0.0041 (8)	−0.0018 (7)
C9	0.0490 (9)	0.0471 (7)	0.0613 (10)	0.0058 (7)	−0.0018 (8)	−0.0009 (7)
C10	0.0527 (11)	0.0570 (9)	0.0816 (13)	0.0005 (8)	−0.0025 (10)	0.0039 (10)
C11	0.0528 (11)	0.0695 (11)	0.1048 (17)	0.0025 (9)	−0.0140 (12)	−0.0067 (12)
C12	0.0711 (14)	0.0692 (11)	0.0837 (14)	0.0144 (11)	−0.0227 (12)	−0.0005 (11)
C13	0.0706 (13)	0.0599 (10)	0.0678 (12)	0.0109 (9)	−0.0078 (10)	0.0059 (9)
C14	0.0559 (10)	0.0478 (7)	0.0586 (10)	0.0070 (7)	−0.0011 (8)	−0.0010 (7)
C15	0.0604 (11)	0.0620 (10)	0.0652 (11)	0.0010 (8)	0.0044 (9)	0.0072 (9)
N1	0.0483 (8)	0.0525 (7)	0.0583 (8)	0.0029 (6)	−0.0018 (7)	0.0025 (6)
O1	0.0530 (8)	0.0886 (9)	0.0596 (8)	0.0050 (7)	0.0044 (7)	0.0051 (7)
F1	0.0835 (10)	0.0967 (9)	0.0861 (9)	−0.0323 (8)	0.0002 (8)	−0.0127 (7)
F2	0.0565 (7)	0.0926 (8)	0.1124 (11)	0.0123 (7)	0.0131 (7)	0.0079 (8)
F3	0.0857 (9)	0.0928 (8)	0.1004 (10)	−0.0116 (8)	0.0083 (8)	0.0376 (8)

### Geometric parameters (Å, °)

**Table:**

C1—C6	1.400 (3)	C9—C10	1.392 (3)
C1—C2	1.411 (3)	C9—C14	1.397 (2)
C1—C8	1.440 (3)	C9—N1	1.412 (2)
C2—O1	1.351 (2)	C10—C11	1.371 (3)
C2—C3	1.398 (3)	C10—H10	0.9300
C3—C4	1.385 (3)	C11—C12	1.377 (3)
C3—C7	1.491 (3)	C11—H11	0.9300
C4—C5	1.376 (3)	C12—C13	1.373 (3)
C4—H4	0.9300	C12—H12	0.9300
C5—C6	1.366 (3)	C13—C14	1.385 (3)
C5—H5	0.9300	C13—H13	0.9300
C6—H6	0.9300	C14—C15	1.484 (3)
C7—H7A	0.9600	C15—F2	1.328 (2)
C7—H7B	0.9600	C15—F1	1.336 (2)
C7—H7C	0.9600	C15—F3	1.343 (2)
C8—N1	1.283 (2)	O1—H1	0.93 (3)
C8—H8	0.9300		
			
C6—C1—C2	118.34 (18)	C10—C9—C14	118.66 (18)
C6—C1—C8	119.82 (18)	C10—C9—N1	122.19 (17)
C2—C1—C8	121.82 (16)	C14—C9—N1	119.10 (17)
O1—C2—C3	117.71 (18)	C11—C10—C9	120.5 (2)
O1—C2—C1	121.19 (17)	C11—C10—H10	119.8
C3—C2—C1	121.10 (18)	C9—C10—H10	119.8
C4—C3—C2	117.4 (2)	C10—C11—C12	120.6 (2)
C4—C3—C7	122.4 (2)	C10—C11—H11	119.7
C2—C3—C7	120.1 (2)	C12—C11—H11	119.7
C5—C4—C3	122.6 (2)	C13—C12—C11	119.8 (2)
C5—C4—H4	118.7	C13—C12—H12	120.1
C3—C4—H4	118.7	C11—C12—H12	120.1
C6—C5—C4	119.7 (2)	C12—C13—C14	120.4 (2)
C6—C5—H5	120.2	C12—C13—H13	119.8
C4—C5—H5	120.2	C14—C13—H13	119.8
C5—C6—C1	120.9 (2)	C13—C14—C9	120.02 (19)
C5—C6—H6	119.6	C13—C14—C15	120.06 (17)
C1—C6—H6	119.6	C9—C14—C15	119.91 (16)
C3—C7—H7A	109.5	F2—C15—F1	106.04 (18)
C3—C7—H7B	109.5	F2—C15—F3	105.81 (17)
H7A—C7—H7B	109.5	F1—C15—F3	105.28 (16)
C3—C7—H7C	109.5	F2—C15—C14	113.09 (15)
H7A—C7—H7C	109.5	F1—C15—C14	113.39 (17)
H7B—C7—H7C	109.5	F3—C15—C14	112.55 (17)
N1—C8—C1	122.48 (18)	C8—N1—C9	120.06 (16)
N1—C8—H8	118.8	C2—O1—H1	105.5 (18)
C1—C8—H8	118.8		
			
C6—C1—C2—O1	−179.82 (16)	C9—C10—C11—C12	−0.5 (3)
C8—C1—C2—O1	2.1 (2)	C10—C11—C12—C13	1.2 (3)
C6—C1—C2—C3	0.7 (2)	C11—C12—C13—C14	−0.7 (3)
C8—C1—C2—C3	−177.39 (15)	C12—C13—C14—C9	−0.4 (3)
O1—C2—C3—C4	−179.06 (16)	C12—C13—C14—C15	179.73 (18)
C1—C2—C3—C4	0.5 (2)	C10—C9—C14—C13	1.1 (2)
O1—C2—C3—C7	1.1 (3)	N1—C9—C14—C13	178.90 (16)
C1—C2—C3—C7	−179.41 (17)	C10—C9—C14—C15	−179.04 (17)
C2—C3—C4—C5	−1.2 (3)	N1—C9—C14—C15	−1.3 (2)
C7—C3—C4—C5	178.6 (2)	C13—C14—C15—F2	−116.15 (19)
C3—C4—C5—C6	0.8 (3)	C9—C14—C15—F2	64.0 (2)
C4—C5—C6—C1	0.4 (3)	C13—C14—C15—F1	123.08 (19)
C2—C1—C6—C5	−1.1 (3)	C9—C14—C15—F1	−56.8 (2)
C8—C1—C6—C5	176.98 (17)	C13—C14—C15—F3	3.7 (3)
C6—C1—C8—N1	−176.38 (16)	C9—C14—C15—F3	−176.13 (16)
C2—C1—C8—N1	1.6 (3)	C1—C8—N1—C9	175.06 (14)
C14—C9—C10—C11	−0.7 (3)	C10—C9—N1—C8	−39.7 (2)
N1—C9—C10—C11	−178.38 (17)	C14—C9—N1—C8	142.65 (17)

### Hydrogen-bond geometry (Å, °)

**Table:**

*D*—H···*A*	*D*—H	H···*A*	*D*···*A*	*D*—H···*A*
O1—H1···N1	0.93 (3)	1.77 (3)	2.619 (2)	151 (3)
C13—H13···F3	0.93	2.36	2.694 (3)	101
